# Is there a role for glucagon‐like peptide‐1 receptor agonists in the treatment of male infertility?

**DOI:** 10.1111/andr.13015

**Published:** 2021-05-05

**Authors:** Rossella Cannarella, Aldo E. Calogero, Rosita A. Condorelli, Emanuela A. Greco, Antonio Aversa, Sandro La Vignera

**Affiliations:** ^1^ Department of Clinical and Experimental Medicine University of Catania Catania Italy; ^2^ Department of Health Sciences University Magna Graecia of Catanzaro Catanzaro Italy; ^3^ Department of Experimental and Clinical Medicine University Magna Graecia of Catanzaro Catanzaro Italy

**Keywords:** GLP1‐RA, hypogonadism, infertility, spermatozoa

## Abstract

Glucagon‐like peptide‐1 receptor agonists (GLP1‐RAs) are novel anti‐hyperglycemic drugs efficacious on glucose control, weight loss, and cardiovascular prevention. These drugs may also be effective in modulating testicular function. In fact, they increase serum testosterone levels in diabetic and/or obese patients with functional hypogonadism on a dysmetabolic basis. Although part of this effect can be ascribed to weight loss, some evidence suggests that there is a direct effect at the testicular level. Indeed, human Leydig, Sertoli, and germ cells express GLP1 receptors. GLP1‐RAs improve sperm metabolism, motility, and insulin secretion in vitro. Likewise, GLP1‐RAs exert positive effects on the metabolism of human Sertoli cells in vitro. Finally, GLP1 is secreted by mouse Leydig cells and this suggests the presence of a paracrine mechanism by which these cells could support the metabolism of Sertoli cells. Therefore, the widespread use of GLP1‐RAs in clinical practice may reveal an important role in the management of male infertility in obese and/or diabetic patients given the negative impact of these diseases on testicular steroidogenesis and spermatogenesis. This should suggest the design of randomized controlled studies aimed at evaluating the effects of these drugs on testicular function.

Diabetes mellitus (DM) is a widespread disease, with a prevalence of 382 million people worldwide in 2013,^1^ which is expected to reach 592 million by the year 2035.^2^ DM is also one of the leading causes of mortality, with a DM‐related death of about 1.3 million in 2013.^2^ To face this issue, novel anti‐hyperglycemic drugs have been developed and introduced in clinical practice. These include dipeptidyl peptidase‐4 inhibitors (DDP4i),^3^ sodium‐glucose co‐transporter‐2 (SGLT2) inhibitors, and glucagon‐like peptide‐1 receptor agonists (GLP1‐RAs). Meta‐analytic data indicate their efficacy in the improvement of glucose control and the prevention of major acute cardiovascular events.^4^, ^5^ Accordingly, the American Diabetes Association (ADA) has recognized their role in the management of type 2 DM (DM2) when metformin alone is not effective in reaching glycemic targets.^3^

SGLT2 inhibitors exert their anti‐hyperglycemic properties by inhibiting glucose reabsorption in the proximal renal tubule, thus increasing urinary glucose excretion and lowering blood glucose levels.^6^ The glucose‐lowering action of both GLP1‐RAs and DPP4i is based on their capability to increase the biochemical activity of GLP1, by stimulating its receptor in the case of GLP1‐RAs or by reducing its catabolism in the case of DPP4i. Under physiologic conditions, GLP1 is released from the intestine and can induce glucose‐dependent insulin secretion from pancreatic β‐cells.^7^, ^8^, ^9^ Specifically, GLP‐1 derives from post‐translational processing of proglucagon. Although the *proglucagon* gene is expressed in enteroendocrine L‐cells and pancreatic α‐cells,^10^ GLP‐1 is synthesized only by intestinal open‐type L‐cells, predominantly located in the ileum and colon, that are in direct contact with nutrients in the intestinal lumen.^11^ Glucose and fat (unlike proteins) are able to stimulate GLP‐1 secretion from L‐cells, with a biphasic pattern (15–30 min after a meal, followed by a second minor peak at 90–120 min).^12^, ^13^ GLP‐1 also hinders glucagon secretion and reduces gastric emptying, promoting glucose control and weight‐loss.^14^ GLP1‐RAs and DPP4i are incretin‐based drugs and positively influence risk factors for cardiovascular disease.^15^

Male hypogonadism is defined by the presence of total testosterone levels lower than 9.2 nmol/L in at least two blood measurements.^16^ About half (44%) of the patients with DM2 and obesity (the so‐called “diabesity”) have hypogonadism mainly with a central mechanism, because of the altered signaling of insulin and leptin at the level of kisspeptin neurons.^17^ The possible role of the new‐hyperglycemic drugs on testosterone levels and sexual function in patients with DM has recently been reviewed.^18^ Evidence in mice has shown that both DPP4i and SGLT2 inhibitors could be able to improve sexual function by their positive effects on the endothelium [eg, by the increase of nitric oxide levels, by the release of vascular endothelial growth factor (VEGF) which induces vasorelaxation, or by exerting anti‐atherogenic effects].^19^, ^20^, ^21^ In line with these data, treatment for four weeks with empagliflozin in rat model of DM2 with erectile dysfunction significantly improved the erectile response in vivo that to electrical stimulation of the cavernous nerve, compared with untreated rats.^22^ These findings support that empagliflozin has a favorable effect on erectile function. No data are available relative to the impact of DPP4i and SGLT2 inhibitors on sexual function and testosterone levels in patients with DM.

In contrast, evidence on the effects of GLP1‐RAs on human testicular function is available. Jensterle and colleagues reported a significant increase in testosterone and a significant reduction of body weight in male patients with obesity‐associated functional hypogonadism.^23^ Specifically, although one‐third of the patients enrolled in this study were diabetic, the prescribed dose of liraglutide was that approved for obesity (3.0 mg/day), which is higher than that approved for diabetes (1.8 mg/day).^23^ Similar conclusions were reported in a retrospective study,^24^ which included diabetic obese patients with overt hypogonadism and poor response to testosterone and metformin administration. Treatment with liraglutide led to a significant increase in testosterone and a significant decrease in body weight. The increased testosterone levels can be likely ascribed to the weight‐lowering effect of GLP1‐RAs, which enhance the leptin signal and stimulates GnRH neuronal function in the hypothalamus. However, the magnitude of testosterone increase in patients treated with the GLP1‐RA liraglutide has been claimed to be higher than expected for the amount of weight loss.^24^ This suggests a possible direct effect of liraglutide in the modulation of the testicular function, which is further supported by the recent identification of GLP1 receptors (GLP1R) in human healthy (non‐tumoral) Leydig cells.^25^ Notably, the positive effects of GLP1‐RA on the erectile function have also been ascribed to the direct action of these drugs on the endothelium.^26^

Interestingly, the role of GLP1‐RAs may not be confined to the management of hypogonadism in patients with DM and/or obesity, because the GLP1/GLP1R axis seems to influence sperm metabolism.

Spermatogenesis can be affected in both type 1 DM (DM1) and DM2. Accordingly, the expression of genes involved in DNA repair is altered in DM1, thus leading to a high rate of DNA fragmentation,^27^ mitochondrial DNA deletions,^28^, ^29^, ^30^, ^31^ and alteration of the respiratory chain with a consequential decrease of sperm motility.^32^, ^33^ Furthermore, being the sperm plasma membrane and acrosome sensitive to insulin, both insulin resistance (as in DM2) or its deficiency (as in DM1) can lead to impaired spermatogenesis.^34^, ^35^ Moreover, glucose plays an important role in spermatozoon metabolism. Indeed, spermatozoa synthesize ATP through glycolysis, mitochondrial oxidative phosphorylation, or the pentose phosphate pathway.^36^ However, not only glucose but also non‐hexose compounds (eg, citrate, pyruvate, lactate) are substrates that spermatozoa can use to obtain energy.^36^ Notably, glucose uptake is highly deregulated in patients with DM.^36^ Particularly, the impairment of glucose homeostasis leads to depletion of GLUTs, which are membrane channels devoted to glucose transport and are expressed in spermatozoa. Their depletion decreases glucose uptake and reduced glucose intracellular availability, which in turn impair sperm metabolism and ATP production. This leads to a decreased sperm motility, increased oxidative stress (OS), and sperm DNA damage.^36^

GLP1 is a potent regulator of glucose homeostasis. Recently, a study carried out in human spermatozoa showed that the GLP1/GLP1R system can influence their metabolism.^37^ In greater detail, the authors demonstrated that GLP1Rs are expressed in human spermatozoa. In β‐pancreatic cells, GLP1, by interacting with its receptor, activates an intracellular signaling pathway involving 3′,5′‐cyclic adenosine 5′‐monophosphate (cAMP) and protein kinase A (PKA).^38^, ^39^ By incubating spermatozoa with increasing concentrations of the GLP1‐RA exenatide, the authors reported a significant increase in sperm motility and cholesterol efflux at the dose of 300 pM.^37^ They further demonstrated that exenatide stimulates insulin secretion from spermatozoa, and, interestingly, influences glucose metabolism because both the lactate dehydrogenase (LDH)‐derived product and the glucose‐6‐phosphate dehydrogenase (G6PDH) activity increased after incubation with exenatide. Similar to what was described in pancreatic β cells, the PKA was involved in the intracellular signaling of exenatide.^37^ This evidence strongly associates spermatozoon metabolism with that of pancreatic β cells, as both of them are insulin‐sensitive and insulin‐secreting cells, and they are both sensitive to the GLP1/GLP1R system.^37^

According to this line of evidence, a study showed that the administration of exenatide (24 nmol/kg/day for 8 weeks) was able to improve sperm quality in terms of sperm motility, mitochondrial membrane potential, and DNA integrity, in high fat diet‐induced obese mice.^40^ Although the contribution of weight reduction in the results observed cannot be excluded, the expression of GLP1R and the effects of exenatide at the spermatozoon level^37^ suggest a direct role of exenatide in the improvement of sperm quality in mice.

Other evidence supports the functional role of the GLP1/GLP1R system also in testicular somatic cells. GLP1Rs have recently been identified in human Sertoli cells.^41^ The exposure to increasing concentrations of GLP1 was able to influence Sertoli cell metabolism. At the lowest concentration, GLP1 increased the efficiency of LDH, while at the highest concentration it reduced mitochondrial membrane potential and oxidative damage.^41^ This leads to speculate that GLP1‐RAs may impact sperm quality and function not only directly—by triggering GLP1R expression in spermatozoa, but also indirectly—by acting via GLP1Rs present in Sertoli cells. In this context, it is interesting to highlight that mouse Leydig cells secrete GLP1.^42^ Notably, GLP1 null male mice are completely infertile and show an abnormal gonadal development.^42^ Thus, Leydig cell‐derived GLP1 may play a role in testicular development at least in mice. Interestingly, this evidence leads to speculate a paracrine mechanism by which Leydig cells might support Sertoli cell metabolism.

Administration of GLP1 to healthy men does not have any effect on gonadotropin levels^43^ but may reduce the number of testosterone pulses.^44^ So far there is no evidence of its effects on in vivo sperm production. Based on the available in vitro data, it is possible to hypothesize that GLP1/GLP1RAs may improve fertility in patients with DM. In fact, these molecules could have a positive impact on sperm metabolism by directly improving their motility and quality and, speculatively, by acting on Sertoli cells (Figure 1).

**FIGURE 1:**
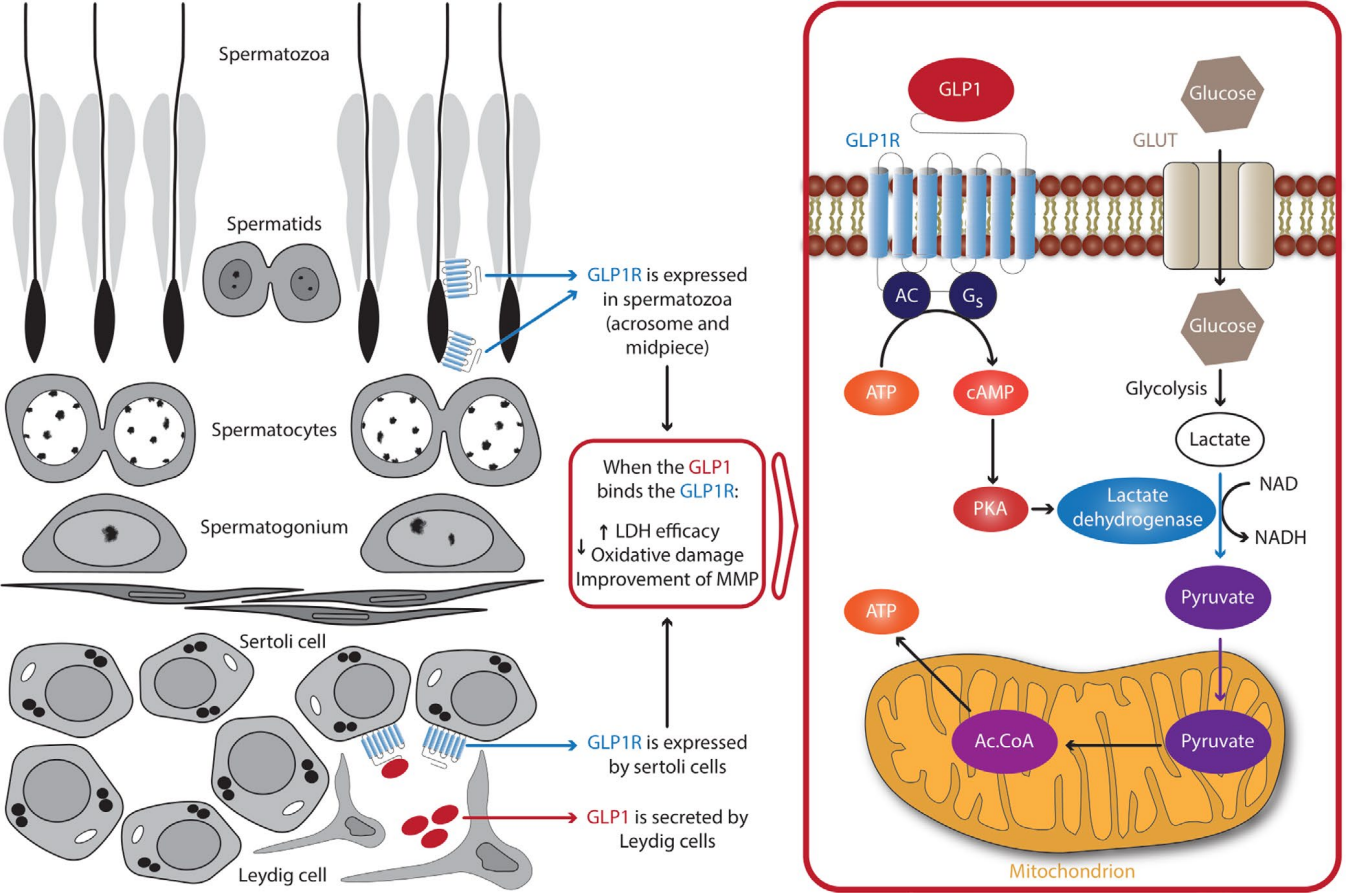
Role of GLP1‐GLP1RA on testicular function. Leydig cells can secrete GLP1, whose receptor is expressed in Sertoli cells and in the sperm midpiece and acrosome. GLP1 could positively impact on the metabolism of these cells, thus improving Sertoli cell function and sperm motility

In conclusion, given the negative impact of DM and obesity on gonadal function in humans, the use of GLP1‐RAs could play an important role in the management of hypotestosteronemia and infertility in patients with these dysmetabolic disorders. At the same time, the evidence reported in this comment should push toward the design of randomized and controlled clinical trials aimed at evaluating the effects of these drugs on the testicular function.

## CONFLICT OF INTEREST

The authors declare no conflict of interest.

## AUTHOR'S CONTRIBUTIONS

Conceptualization: R.C. and S.L.V.; Investigation: R.C.; Methodology: E.G.; Project administration: A.E.C. and S.L.V.; Supervision: R.A.C. and A.A.; Visualization: R.A.C., E.G., A.A., and R.A.C.; Writing—original draft: R.C.; Writing—review and editing: S.L.V. and A.E.C.
